# 2-[^18^ F]fluoro-2-deoxy-d-glucose (FDG) positron-emission tomography (PET) findings of chronic expanding intrapericardial hematoma: a potential interpretive pitfall that mimics a malignant tumor

**DOI:** 10.1186/1749-8090-8-13

**Published:** 2013-01-17

**Authors:** Hiroyuki Tokue, Azusa Tokue, Kenzo Okauchi, Yoshito Tsushima

**Affiliations:** 1 Department of Diagnostic and Interventional Radiology, Gunma University Hospital, 3-39-22 Showa-machi, Maebashi, Gunma, 371-8511, Japan

**Keywords:** 2-[^18^ F] fluoro-2-deoxy- d-glucose (FDG), Positron-emission tomography (PET), Chronic expanding intrapericardial hematoma, Intrapericardial tumor

## Abstract

A 77-year-old man who had undergone mitral valve replacement 5 years previously presented with an intrapericardial mass. Computed tomography and magnetic resonance imaging showed that the mass lesion contained hematoma components. Positron-emission tomography (PET) with 2-[^18^ F] fluoro-2-deoxy-d-glucose (FDG) revealed uptake in the peripheral rim of the mass. These findings suggested the presence of hematoma associated with a malignant lesion. Surgical resection was performed, and the histological diagnosis was chronic expanding intrapericardial hematoma without neoplastic changes. Chronic expanding intrapericardial hematoma is a rare disease but should be considered when an expanding mass is found in a patient after cardiac surgery. The FDG-PET findings of chronic expanding hematomas, including FDG uptake in the peripheral rim of the mass as a result of inflammation, should be recognized as a potential interpretive pitfall that mimics a malignant tumor.

## Background

Chronic expanding intrapericardial hematoma is a rare disease that occurs after open heart surgery, chest trauma, or epicardial injury. Chronic expanding hematomas can be misdiagnosed as malignant tumors because of their large size and slow, progressive enlargement [1-3].

Positron-emission tomography (PET) with 2-^18^ F] fluoro-2-deoxy-d-glucose (FDG) is an evolving diagnostic modality used for tumor detection, staging, therapeutic monitoring, and follow-up of various malignant tumors. We found only a few reports on chronic expanding hematomas, and they had very limited FDG-PET imaging features [4-6].

We present a case of chronic expanding intrapericardial hematoma after cardiac surgery. The lesion exhibited increased activity on FDG-PET, mimicking the characteristics of a malignant lesion.

## Case presentation

A 77-year-old man who had had undergone mitral valve replacement 5 years previously was admitted to our hospital because of chest discomfort. A small mass near the left side of the heart had been detected 2 years earlier by chest roentgenography. The mass had gradually increased in size. The patient had been receiving anticoagulant therapy with warfarin since the previous operation. He had taken an average dose of 10 mg of warfarin daily for 2 years. The international normalized ratio (INR) range was kept at 2.0–2.5. He had no history of pulmonary tuberculosis or chest trauma. Laboratory studies revealed chronic hypochromic anemia, with a hemoglobin level of 11.9 g/dL and hematocrit level of 35.5%. There was no apparent increase in the levels of tumor markers, including carcinoembryonic antigen (CEA), carbohydrate antigen 19–9 (CA19-9), neuron-specific enolase (NSE), squamous cell carcinoma–related antigen (SCC), and interleukin-2 receptor.

Chest roentgenography revealed an enlargement of the left side of the heart.

**Figure 1:**
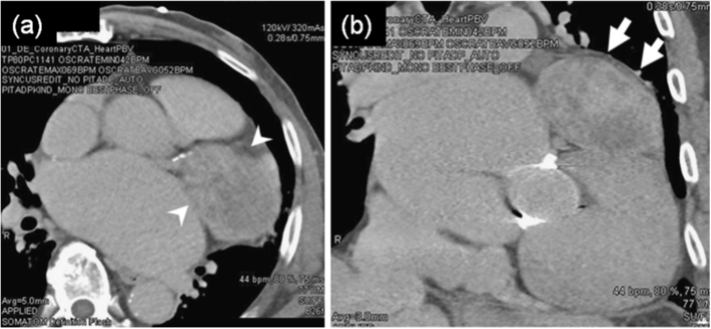
**Chest computed tomography (CT). (a)** A 77-year-old man presented with an intrapericardial mass. Contrast-enhanced CT demonstrated a huge mass adhered to the left atrial appendage. The mass was not uniform; it lacked calcification and showed non-homogeneous enhancement (arrowheads). **(b)** Coronal contrast-enhanced CT shows that the mass was covered with pericardium (arrows).

Contrast-enhanced computed tomography (CT) demonstrated a huge mass adhered to the left atrial appendage and covered with pericardium. The mass was not uniform; it lacked calcification and showed non-homogeneous enhancement. The mass measured 9 × 6 × 4 cm (Figure 1a, 1b).

**Figure 2:**
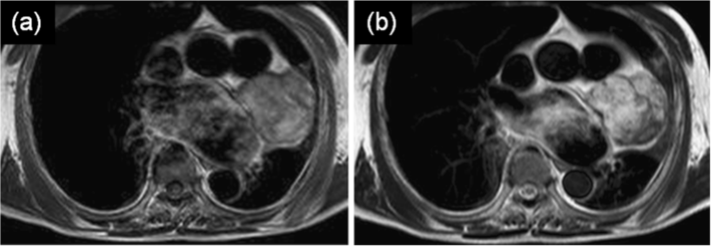
**Magnetic resonance imaging. (a)** A T1-weighted image (T1WI) demonstrated a well-defined mass of slightly high intensity. **(b)** A T2WI demonstrated a mixture of high- and low-intensity areas. A low-intensity septum and a peripheral rim were observed in the mass on both T1WI and T2WI.

On magnetic resonance imaging (MRI), a T1-weighted image (T1WI) demonstrated a well-defined mass of slightly high intensity (Figure 2a). A T2-weighted image (T2WI) demonstrated a mixture of high- and low-intensity areas (Figure 2b). A low-intensity septum and a peripheral rim were observed in the mass on both T1WI and T2WI.

**Figure 3:**
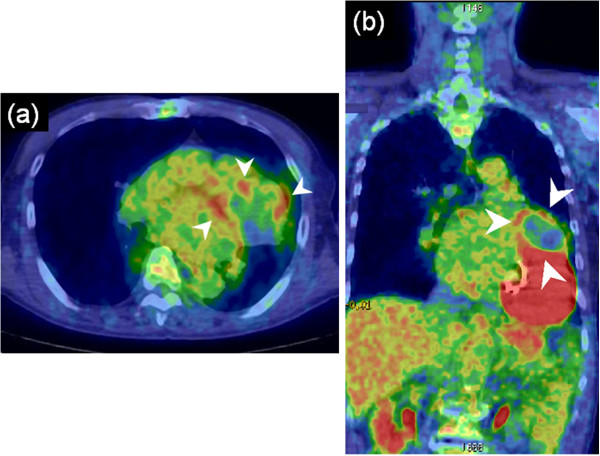
**Fused positron-emission tomography (PET) and computed tomography (CT) image. (a)** Axial fused PET and CT image shows 2-[^18^ F] fluoro-2-deoxy-d-glucose (FDG) uptake in the peripheral rim of the mass (arrowheads). The maximum standardized uptake value (SUV) of this lesion was 3.50. **(b)** Coronal fused PET and CT image. Other signs of abnormal uptake suggesting a malignant lesion were not observed in other intrapericardial mass (arrowheads).

FDG-PET images revealed uptake in the peripheral rim of the mass (Figure 3a, 3b). The maximum standardized uptake value (SUV) of this lesion was 3.50. Other signs of abnormal uptake suggesting a malignant lesion were not observed. The patient fasted for 6 h before receiving an intravenous injection of 18 F-FDG (5 MBq/kg). FDG PET/CT scans were obtained using Biograph 16 (Siemens Medical Solutions; Knoxville, TN, USA) scanners, with a 700-mm field of view (FOV) and a slice thickness of 3.27 mm. The CT was acquired to correct PET transmission using the following parameters: 140 kV and 120–240 mAs to produce 128 × 128 matrix images. The patient was scanned in the arms-down position, from head to thigh. Shallow breathing was advised to avoid motion artifacts and minimize misregistration of CT and PET images. Intravenous contrast material was not administered for CT scanning. After the CT scan, the PET data were acquired, and acquisition time was 3 min per bed position. CT images were reconstructed using the conventional filtered back-projection method. Axial full width at half-maximum at 1 cm from the center of the FOV was 6.3 mm.

The findings were interpreted as being suggestive of hematoma associated with a malignant lesion. We could not rule out a pericardial or mediastinal malignant tumor with bleeding.

**Figure 4:**
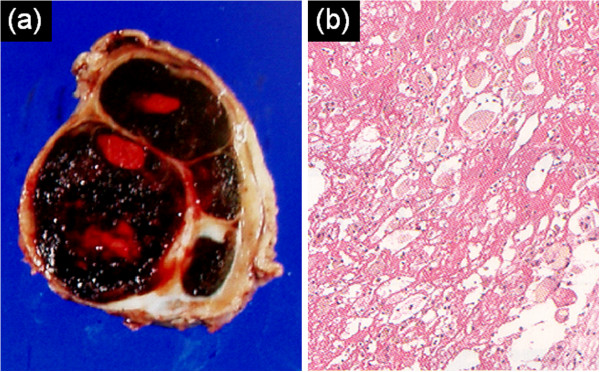
**Macroscopic and pathological findings. (a)** Macroscopic observations showed that the resected mass consisted of a dark red, partially organized hematoma containing a small amount of liquid with a fibrous membrane. **(b)** Pathological examination showed a hematoma surrounded by dense fibrous tissues, and the center of the hematoma consisted of fresh and old hemorrhages. Focal infiltration of hemosiderin-laden macrophages was observed in the outer zone of the peripheral wall. No malignant change was observed.

After obtaining informed consent, we performed surgical resection and found an elastic, hard mass covered with pericardium. The mass was widely adhered to the left atrial appendage, which was carefully detached. Partial pericardiectomy and complete removal of the mass were successful. The location of the mass was distinct from the anastomosis site in the left circumflex artery, and the source of bleeding seemed to be the branch of the left coronary artery. Rapid pathological examination identified a hematoma without neoplastic changes. Macroscopic observations showed that the resected mass consisted of a dark red, partially organized hematoma containing a small amount of liquid with a fibrous membrane (Figure 4a). The result of bacterial culture was negative. Pathological examination showed a hematoma surrounded by hyaline fibrous tissues, and the center of the hematoma consisted of fresh and old hemorrhages. Focal infiltration of hemosiderin-laden macrophages was observed in the outer zone of the peripheral wall. No malignant change was observed (Figure 4b). This confirmed a diagnosis of chronic expanding intrapericardial hematoma. From the histological viewpoint, the low-intensity septum and a peripheral rim on both T1WI and T2WI corresponded with the pseudocapsule of hyaline fibrous tissue. The mixture of high- and low-intensity areas on T2WI corresponded with fresh and old hemorrhages. The area of focal infiltration of hemosiderin-laden macrophages was consistent with high FDG uptake in the peripheral area of the mass.

The patient had an uneventful postoperative recovery, without complications. His chest discomfort was alleviated. Approximately 2 years after the operation, there is no sign of recurrence.

## Discussion

Chronic expanding intrapericardial hematoma is particularly rare. According to Reid et al., a hematoma that persists and increases in size more than 1 month after the initial hemorrhagic event is a chronic expanding hematoma [1]. In most cases, soft tissue hematomas resolve spontaneously. The mechanism of expansion of such hematomas is still incompletely understood. However, they have developed in regions of previous trauma and surgery in patients with hemorrhagic diathesis and those receiving anticoagulant therapy; these hematomas could also occur spontaneously or be caused by a minor or unappreciable trauma [7]. The irritation releases vasoactive substances and induces capsule formation, while repeated inflammation seems to result in effusion and new bleeding from damaged microvessels beneath the capsule [8].

The radiological appearance of a chronic expanding hematoma varies. The CT findings consist of a heterogeneous mass with a wall of variable thickness that often contains peripheral areas of calcification [9]. On MRI, a chronic expanding hematoma has been reported to have a low-signal-intensity peripheral capsule and central contents with signal intensities ranging from high to low, a so-called mosaic sign. The various signal intensities indicate the presence of fresh and old blood, caused by repeated bleeding over time. MRI is very important in preoperative diagnosis, and histopathological examination is crucial for differential diagnosis with soft tissue sarcomas [5].

FDG-PET images of chronic expanding hematoma are not widely available. Only 4 reports, including the present one, about FDG-PET imaging features of chronic expanding hematomas have been documented in the English-language literature (Table 1). In previous reports, the peripheral portion of the chronic expanding hematoma tended to take up FDG [4-6]. Only 1 other article has presented FDG-PET imaging features of brain hematomas [10], which appeared as scattered foci of increased FDG uptake around a hematoma.

**Table 1:** Documented cases of chronic expanding hematomas and their FDG-PET characteristics

**First Author**	**Age, years**	**Sex**	**Clinical Presentation**	**Location**	**Size, cm**	**History**	**SUV**
Hamada [4]	65	M	femoral neuropathy	right ilium	8 × 5	no history	3.1
Kwon [5]	67	F	dyspnea	right hemithorax	NS	pneumonectomy for pulmonary tuberculosis	3.7
Takahama [6]	77	M	intermittent pain	right chest wall	4.5 × 2.2	tuberculous pleurisy	5.5
Tokue (present)	77	M	chest discomfort	intrapericardial	9 × 6 × 4	mitral valve replacement	3.5

Abbreviations: FDG, 2-[^18^ F] fluoro-2-deoxy-d-glucose; NS, data not shown; PET, positron-emission tomography; SUV, standardized uptake value

In all cases, the peripheral portion of the chronic expanding hematoma tended to take up FDG.

To the best of our knowledge, FDG-PET images of a chronic expanding intrapericardial hematoma have not been previously reported. We observed increased FDG uptake in the peripheral rim of the mass in our patient.

FDG-PET imaging is increasingly being used in clinical oncology because it enables functional imaging of various tumors. Generally, high-grade sarcomas and aggressive benign lesions have higher SUVs than do benign lesions. However, the use of FDG-PET imaging for tumor diagnosis is limited by the fact that FDG, a glucose analog, is taken up not only by tumor cells but also by macrophages and tissue with granulation and inflammation [4,6]. High uptake of FDG has been observed in many types of inflammatory lesions. A previous autoradiographic study demonstrated that macrophages and immature granulation tissue containing fibroblasts contribute to the increased FDG uptake in tumors [4,6]. In our case, FDG uptake was observed in the peripheral rim, which contained hemosiderin-laden macrophages. This inflammatory reaction likely caused the positive uptake of FDG. FDG uptake in the peripheral rim is not a specific sign of hematoma. The same pattern might be seen if a malignant tumor has a tendency of central necrosis. However, the characteristics of FDG-PET images of chronic expanding hematomas, including the uptake of FDG in the peripheral rim of the mass as a result of inflammation, should be recognized as a potential interpretive pitfall that mimics a malignant tumor.

Such hematomas should be managed with complete surgical resection at an early stage, before cardiac and mediastinal compression or extrathoracic protrusion occur [5].

## Conclusion

In summary, we have presented FDG-PET findings of a chronic expanding intrapericardial hematoma with SUVs that could have caused an interpretive pitfall by mimicking a malignant tumor. Chronic expanding intrapericardial hematoma is a rare disease but should be considered when an expanding mass is found in a patient after cardiac surgery.

## Consent

Written informed consent was obtained from the patient for publication of this Case report and any accompanying images. A copy of the written consent is available for review by the Editor-in-Chief of this journal.

## Competing interests

The authors declare that they have no competing interests.

## Authors’ contribution

All authors read and approved the final manuscript.
